# The Multiplicity of Infection-Dependent Effects of Recombinant Adenovirus Carrying HGF Gene on the Proliferation and Osteogenic Differentiation of Human Bone Marrow Mesenchymal Stem Cells

**DOI:** 10.3390/ijms19030734

**Published:** 2018-03-05

**Authors:** Qian Wen, Shimeng Zhang, Xialin Du, Ruining Wang, Yanfen Li, Honglin Liu, Shengfeng Hu, Chaoying Zhou, Xinying Zhou, Li Ma

**Affiliations:** Institute of Molecular Immunology, School of Laboratory Medicine and Biotechnology, Southern Medical University, Guangzhou 510515, China; wencaoxi@smu.edu.cn (Q.W.); sz144@duke.edu (S.Z.); DUXIALIN@outlook.com (X.D.); wrn470242269@163.com (R.W.); 18926214619@163.com (Y.L.); 18638875241@163.com (H.L.); hushengfeng@smu.edu.cn (S.H.); zhouchao@smu.edu.cn (C.Z.); zxyforever@smu.edu.cn (X.Z.)

**Keywords:** avascular necrosis of femoral head, human bone marrow mesenchymal stem cell, hepatocyte growth factor, adenovirus, signaling pathway, proliferation, osteogenesis

## Abstract

Absence of effective therapeutic methods for avascular necrosis of femoral head (ANFH) is still perplexing the world’s medical community. Bone marrow mesenchymal stem cells (BMSCs) adoptive cell therapy combined with core decompression is a promising modality, which is highly dependent on the cellular activities of BMSCs. Hepatocyte growth factor (HGF) is a survival factor for BMSCs, yet the underlying mechanism is not fully elucidated. In this study, the effects of multiplicity of infections (MOIs) of recombinant adenovirus carrying HGF gene (rAd-HGF) on human BMSC proliferation and osteogenic differentiation were systemically examined. Infection of rAd-HGF produced secretory HGF and promoted hBMSC proliferation in a MOI-dependent manner, while the osteogenesis was also strengthened as indicated by enhanced calcium nodule formation with the strongest effects achieved at MOI = 250. Blocking the activities of c-MET or its downstream signaling pathways, WNT, ERK1/2, and PI3K/AKT led to differential consequents. Specifically, blockage of the WNT pathway significantly promoted osteogenic differentiation, which also showed additive effects when combined application with rAd-HGF. Our data demonstrated the pro-osteogenic effects of optimized MOIs of rAd-HGF, while inhibition of WNT pathway or activation of PI3K/AKT pathway may act as candidate adjuvant modalities for promoting osteogenic differentiation in rAd-HGF-modified hBMSC treatment on ANFH.

## 1. Introduction

Avascular necrosis of the femoral head (ANFH) is a common and frequently-occurring disease, mainly occurring in adults aged 20–50 years old. The disability rate of ANFH is extremely high, seriously affecting the life quality and working capacity of patients, resulting in a huge burden for society and families. Around 30 million people worldwide are suffering from the disease, and the incidence is still growing.

Currently, there is no effective cure for ANFH [1], and this has become the challenge to be jointly tackled by the world’s medical community. Interventional therapies, medullary core decompression, vascularized bone graft surgery, osteotomy, etc., have been used at the early stages (Ficat stage I and II), yet nearly all of them could not stop the development of disease. When advanced to stage III and IV, femoral head collapse could occur, and can be only rescued by artificial joint replacement, but with a failure rate of 10–50% within five years. Due to most people by this disease mainly being young adults, and by far the best artificial femoral head can only be used for 10–15 years, patients often must experience two or three replacements, which is a painful process with a heavy financial burden. Therefore, exploring new and effective early treatment and trying the best to preserve the patient’s own femoral head, is especially important for ANFH therapy. The bone marrow mesenchymal stem cell (BMSC) adoptive cell therapy has been extensively tested in clinical, and the improvement of the curative effect has been well recognized. BMSCs are injected through the hole resulting from the core decompression of the necrotic femoral head to take part in bone repair and regeneration [1], on the one hand by secreting hepatocyte growth factor (HGF) and other nutritional factors, on the other hand by proliferating and differentiating into osteogenic cells in the local bone microenvironment composed by the bone marrow and the trabeculae of cancellous bone [2]. Farzan M et al. compared the therapeutic efficacy of core decompression with or without BMSC transplantation in 28 hips with early-stage ANFH using the WOMAC (Western Ontario and McMaster Universities Osteoarthritis Index) questionnaire, VAS (visual analogue scale) pain index, and magnetic resonance imaging (MRI). Results showed that all patients achieved improved condition significantly (*p* < 0.001) assessed by the mean WOMAC and VAS scores, while MRI examination indicated a significant improvement in hips with bone marrow stem cell injection (*p* = 0.046) and significant worsening in hips without stem cell injection (*p* < 0.001), confirming the effectiveness of using BMSCs in treatment of early ANFH [3].

The exact mechanism of tissue repair mediated by BMSCs is not fully elucidated, but studies have shown that BMSC-secreted HGF and other neurotrophic factors have played important roles in this process [4]. HGF is a pleiotropic cytokine and functions as a potent mitogen and histologic nutrition factor to promote repair in adult tissues, such as the liver [5], heart [6], and muscle [7]. In addition, HGF also has pro-angiogenic effect that can improve the underlying cause of ANFH—the blood disorder—while inhibiting apoptosis and reducing fibrosis [8,9]. In vivo, HGF gene-modified BMSCs significantly accelerated the myocardial tissue repair of myocardial infarction after trauma compared with un-transfected BMSCs [10], while BMSCs with HGF gene knocked out could not improve blood vessel regeneration in the limb ischemia model [11]. We verified the therapeutic efficacy of HGF gene-modified BMSC treatment on ANFH in the rabbit model, and further explored the mechanisms. Our data indicated the difference in the activation of downstream different signaling pathways of the HGF receptor c-MET by different doses of HGF, resulting in distinct biological effects: in osteogenic environment, 100 ng/mL of HGF induced higher level of ERK1/2 pathway activation, which can significantly promote BMSC proliferation and inhibit osteogenic differentiation; while 20 ng/mL of HGF activated the AKT pathway with higher level than its effects on the ERK1/2 pathway activation, thus prompting BMSCs to differentiate into osteoblasts. This change in exogenous expression of HGF made genetically-modified BMSCs show different characteristics of life activities at different stages of treatment, at early stage cells bloomed so as to achieve the number required for organization repair, then differentiated into osteogenic cells in the bone microenvironment, playing an effective role in tissue repair [12]. However, whether the above founding in rabbit BMSCs also works with the similar effects in human BMSCs is unknown.

C-MET is a receptor tyrosine kinase (RTKs) and essential for embryonic development and wound healing, with HGF as the only known ligand. Activation of downstream signal pathways mediated different biological effects of c-MET. Among them, the RAS pathway mediates HGF-induced cell dispersion and proliferation signals, resulting in branching morphogenesis [13]. Different from most mitogens, HGF induces sustained RAS activation, and thus prolonged activation of MAPK, together with the STAT pathway played a key role in mediating cell invasive growth and morphogenetic processes [14]. Activation of PI3K pathway is through two approaches: it is downstream molecules of RAS, and also directly recruited by the cohesion protein binding sites on the c-MET [15]. PI3K activation also triggers a survival signal activated by the AKT pathway activation [16]. C-MET downstream signal pathways also include the β-catenin pathway. The β-catenin pathway is a classic key member of the WNT signaling pathway, translocating into the nucleus following c-MET activation and involving in the transcriptional regulation of a large number of genes [17]. Classic WNT signaling pathway promotes osteoblastic differentiation of BMSCs early in osteogenesis by relying on or not relying on the osteogenic differentiation of key transcription factor runt-related transcription factor 2 (Runx2) [18]. Clearly, each of signal pathways coordinated the control on cell activity. Therefore, fully aware of the effects of different HGF levels on the c-MET downstream signaling pathways related to osteogenic differentiation and proliferation will provide theoretical support for a more rational use of HGF to promote BMSC effect on bone tissue repair in osteogenic environments.

In this study, we generated HGF-gene modified hBMSCs using rAd-HGF and tested the effects of low MOI (MOI = 10 or 50) and high MOIs (MOI = 250 or 1250) on the expression of HGF which was thought to be critical regulator in ANFH therapy. Our results indicated that higher MOIs of rAd-HGF (MOI = 250 or 1250) not only significantly promote HGF expression, but also play stronger roles in enhancing hBMSC proliferation and osteogenic differentiation than rAd-Ctrl and low MOIs of rAd-HGF (MOI = 10 or 50). Inhibitor-specific blockage of signaling pathways showed that the WNT signaling pathway plays anti-osteogenic roles while the PI3K/AKT pathway is pro-osteogenic for hBMSCs. Our findings not only indicated the optimized MOI of rAd-HGF for preparing HGF-gene modified hBMSCs, but also suggested that the WNT pathway is a promising inhibitory target for treatment of ANFH using rAd-HGF-modified hBMSCs.

## 2. Results

### 2.1. The Effects of rAd-HGF MOI on the Expression of HGF in hBMSCs

**Figure 1:**
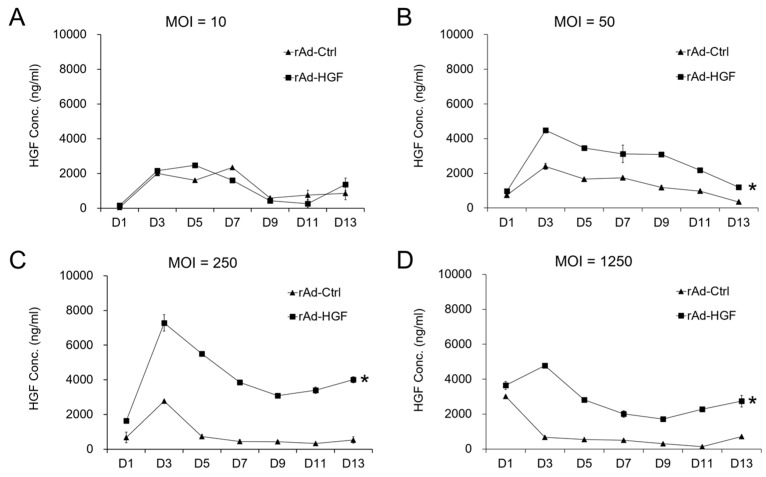
Secretion of HGF by hBMSCs infected with recombinant adenoviruses. Human BMSCs were cultured in the growth medium and infected with rAd-HGF or the control recombinant adenovirus rAd-Ctrl at MOI = 10 (**A**), 50 (**B**), 250 (**C**), and 1250 (**D**), and the secretory HGF level was assayed using ELISA. The results showed that rAd-HGF infection significantly promoted HGF secretion starting with MOI = 50. * *p* < 0.05. The experiment has been replicated three times with similar results, and the representative results were shown.

Human BMSCs naturally express HGF in a relatively low level [19]. To explore whether the expression levels of HGF in hBMSCs from rAd-HGF infection are dependent on the MOI, the concentrations of secretory HGF protein in the growth medium of hBMSCs infected by serial MOIs of rAd-HGF were detected by ELISA every two days for about two weeks. As predicted, control adenovirus (rAd-Ctrl)-infected hBMSCs maintained the secretion of HGF, while low MOI (MOI = 10) of rAd-HGF brought out the similar HGF concentrations during a 13-day observation period (Figure 1A). In contrast, significant increase in HGF secretion compared with the rAd-Ctrl infection group could be observed in higher MOI (MOI = 50) of rAd-HGF infection (*p* < 0.05; Figure 1B). The peak concentration of HGF in the supernatants was detected at MOI = 250 (Figure 1C), and the significant upregulation of secretory HGF concentrations was maintained with ascending MOIs to the highest MOI detected in this study (MOI = 1250; Figure 1D). The above results indicated that overexpression of HGF could be achieved by infection of hBMSCs with higher MOIs (MOI ≥ 50) of rAd-HGF.

### 2.2. Evaluating the Pro-Proliferative Effects of Serial rAd-HGF MOIs on hBMSCs

**Figure 2:**
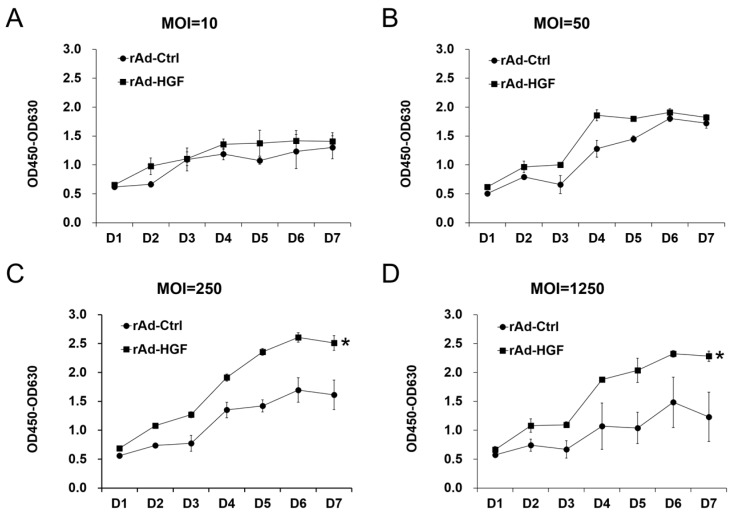
Effects of rAd-HGF on hBMSC proliferation. Human BMSCs were cultured in the growth medium and infected with rAd-HGF or rAd-Ctrl at MOI = 10 (**A**), 50 (**B**), 250 (**C**), and 1250 (**D**). The WST-8 method was used to evaluate the proliferation level. Infection with rAd-HGF showed significant promotive effects on hBMSC proliferation starting with MOI = 250. * *p* < 0.05. The experiment has been replicated four times with similar results, and the representative results were shown.

To investigate the influence of serial MOIs of rAd-HGF infection on hBMSC proliferation, the cell viability analysis was performed following rAd-HGF infection. Results of the WST-8 assays indicated that in growth medium, relative lower doses of HGF obtained by rAd-HGF infection at MOI = 10 or 50 could not significantly promote cell growth compared with the effects of rAd-Ctrl. At MOI = 10, there was no difference in proliferation between cells infected with rAd-HGF and those infected with rAd-Ctrl (Figure 2A). Although there was some elevation in cell amounts resulting from infection with rAd-HGF compared with rAd-Ctrl at MOI = 50, such a significant difference disappeared beginning at five days post infection (Figure 2B). However, the enhanced cell proliferation became more significant starting from the second day after higher MOIs (MOI = 250 or 1250) of rAd-HGF infection, which is probably owing to the higher HGF expression induced by rAd-HGF infection with the higher MOIs. Such pro-proliferative effects became more remarkable at the later timepoints of the observation period (Figure 2C,D). Consistent with the secretion of HGF, the highest level of cell proliferation was observed in cells infected with rAd-HGF at MOI = 250 (Figure 2C). 

### 2.3. hBMSC Osteogenic Differentiation Induced by Serial MOIs of rAd-HGF Infection

**Figure 3:**
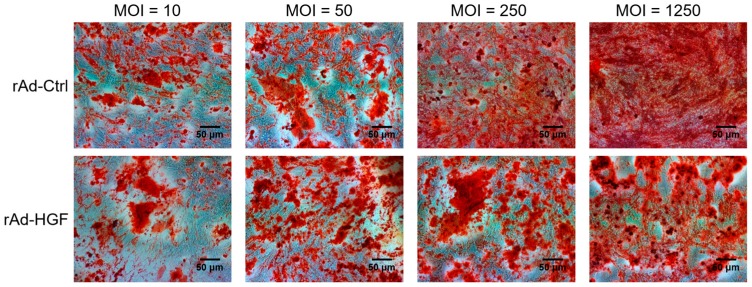
Effects of HGF overexpression on hBMSC osteogenesis. Human BMSCs were cultured in the osteogenic differentiation medium and infected with rAd-HGF or rAd-Ctrl at MOI = 10, 50, 250, and 1250. AR-S staining was used to assess the osteogenic level of hBMSCs. Results showed that osteogenesis occurred in cells infected with rAd-Ctrl or rAd-HGF, while overexpression of HGF further promoted such differentiation. The experiment has been replicated three times with similar results, and the representative results were shown.

To study the MOI influence of rAd-HGF infection on hBMSC differentiation, the osteogenesis was induced by changing the growth medium to differentiation medium and then hBMSCs were infected by serial MOIs of rAd-HGF. Two weeks later, the degree of osteogenic differentiation indicated by calcium accumulation was evaluated using AR-S staining. Most hBMSCs infected with a relatively high-level of rAd-Ctrl spread relatively evenly in the culture surface, accompanied with the relatively even AR-S staining. In contrast, rAd-HGF-infected cells with AR-S positive staining were clustered together to form calcium nodules that distributed unevenly. In the central of the calcium nodules formed by hBMSCs infected with rAd-HGF but not rAd-Ctrl, AR-S staining showed deep to dark color which indicated higher expression of osteocalcin. The results showed that osteogenic differentiation of hBMSCs gradually enhanced along with the ascending MOIs of rAd-HGF as indicated by the remarkable increase in the formation of mineralized extracellular matrix (ECM). Cells infected with rAd-HGF at MOI = 10 or 50 showed no apparent difference of mineralized ECM formation compared with rAd-Ctrl-infected cells. However, when MOI elevated to 250 or 1250, we observed significantly enhanced osteogenic differentiation in hBMSCs infected with rAd-HGF than in those infected with rAd-Ctrl (Figure 3).

### 2.4. Roles of Different Signaling Pathways in hBMSC Proliferation Induced by rAd-HGF Infection

**Figure 4:**
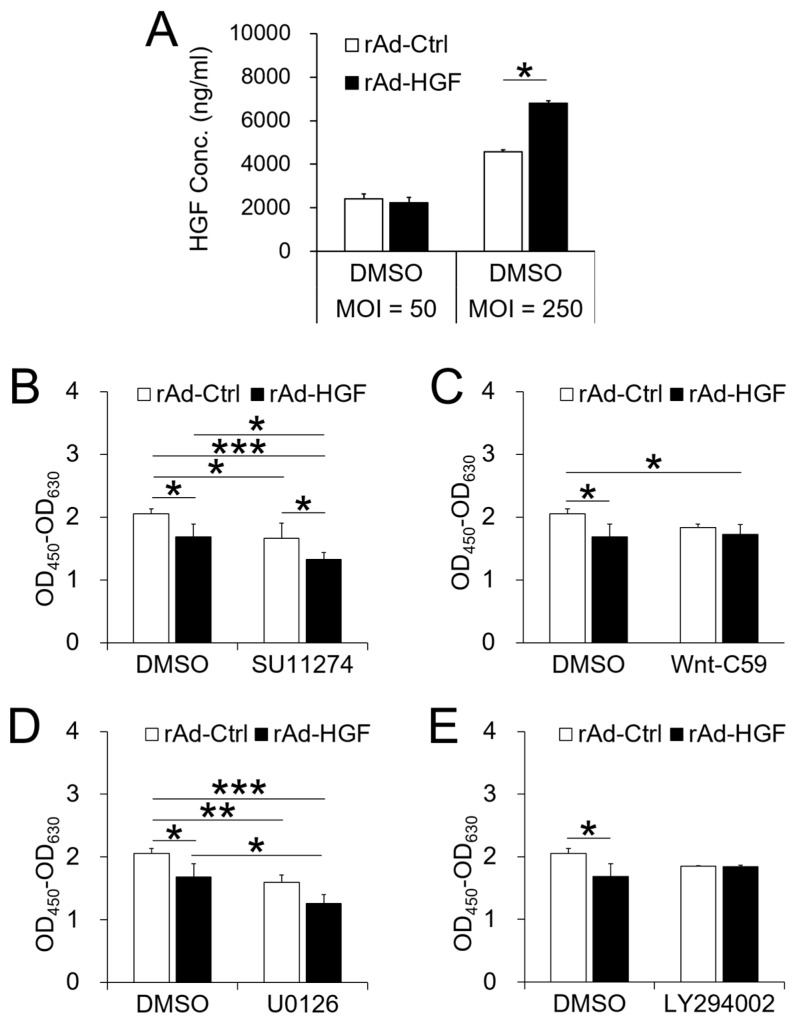
Roles of signaling pathways in HGF secretion and cell proliferation of hBMSCs infected with recombinant adenoviruses. Human BMSCs were cultured in the osteogenic differentiation medium and treated with various signal pathway inhibitors or DMSO before infection. Three days later, HGF secretion by cells treated with DMSO were evaluated using ELISA (**A**), and cell proliferation was detected using the WST-8 method (**B**–**E**). * *p* < 0.05, ** *p* < 0.01, *** *p* < 0.001. The experiment has been replicated three times with similar results, and the representative results were shown.

Our previous studies have shown that ERK1/2 and PI3K/AKT signaling pathways are involved in regulating the proliferation and osteogenesis of BMSCs [12]. The WNT signaling pathway was reported to regulate the transcription of many genes, among them, the induction of RUNX2 promotes BMSC osteogenic differentiation [18]. To explore the roles of signaling pathways on the hBMSC proliferation and osteogenic differentiation induced by rAd-HGF infection, corresponding inhibitors against the above pathways were used. As various MOIs of rAd-HGF induced significantly different effects on proliferation and osteogenic differentiation in hBMSCs, with the highest levels were observed at MOI = 250, and in the osteogenic differentiation medium, the level of secreted HGF by hBMSCs infected with rAd-HGF was significantly higher than that by cells infected with rAd-Ctrl only at MOI = 250, but not at MOI = 50, as indicated by the results of ELISA (Figure 4A), the impacts of signaling pathways on proliferation and osteogenesis of hBMSCs were evaluated with rAd-HGF infection at this MOI. The inhibitory effects on these signaling pathways have been confirmed using Western blot analysis (Figure S1). The impact of recombinant adenovirus infection combined with inhibitor treatment on cell status was observed under the microscope (Figure S2), and results of WST-8 assays indicated that, in osteogenic differentiation medium, treatment with different inhibitors resulted in various effects on cell proliferation. In contrast to that in the growth medium, rAd-HGF infections led to a significant decrease in the total number of cells in osteogenic differentiation medium with mock treatments of DMSO as controls (Figure 4B–E).

However, using SU11274 to inhibit the HGF receptor, c-MET, significantly suppressed the cell proliferation, no matter whether cells were infected with rAd-HGF or with rAd-Ctrl. Unexpectedly, cells infected with rAd-HGF could not counteract the anti-proliferative effects to c-MET signaling by inhibitor SU11274. In comparison, SU11274 treatment further inhibited the proliferation of cells infected with rAd-HGF, resulting in the lowest level of proliferation in hBMSCs infected with rAd-HGF but not in cells with rAd-Ctrl. This result suggests that HGF-induced cell proliferation is dependent on c-MET activity (Figure 4B).

Meanwhile, roles of other c-MET downstream signaling pathways were detected. For this goal, the WNT inhibitor, Wnt-C59, was used to treat cells before rAd-HGF infection. Unexpectedly, when cells were infected with recombinant adenoviruses, inhibiting the WNT pathway significantly suppressed cell proliferation induced by rAd-HGF infection when compared with the rAd-Ctrl-infected cells treated with DMSO (*p* < 0.05). However, for cells infected with rAd-HGF, there was no significant difference between the proliferation level of cells treated with DMSO and that of cells treated with Wnt-C59. Meanwhile, for cells treated with Wnt-C59, there is no significant difference in proliferation observed between cells infected with rAd-HGF and cells infected with rAd-Ctrl. These results indicated that the WNT pathway did not play important roles in the rAd-HGF infection-induced hBMSC proliferation in the osteogenic medium (Figure 4C). 

Another signaling pathway involved in cell proliferation was the ERK1/2 pathway. Pre-treatment of cells with the corresponding inhibitor U0126 significantly inhibited cell proliferation when compared with DMSO-treated and rAd-Ctrl-infected cells. Meanwhile, U0126 treatment resulted in significant decrease in the proliferation level of cells infected with rAd-HGF compared with rAd-HGF-infected cells treated with DMSO (*p* < 0.05), indicating that HGF induced cell proliferation by activating ERK1/2 signaling pathway downstream of c-MET (Figure 4D). 

Contrary to the ERK1/2 pathway, the PI3K/AKT pathway has been reported to promote rat BMSC osteogenic differentiation [20]. In this study, the role of the PI3K/AKT pathway in proliferation was also detected. As expected, no obvious effect on cell proliferation was observed with LY294002 treatment, suggesting that the PI3K/AKT pathway did not take part in the regulation of hBMSC proliferation in the osteogenic differentiation medium (Figure 4E).

### 2.5. Roles of Different Signaling Pathways in hBMSC Osteogenic Differentiation Induced by rAd-HGF Infection

**Figure 5:**
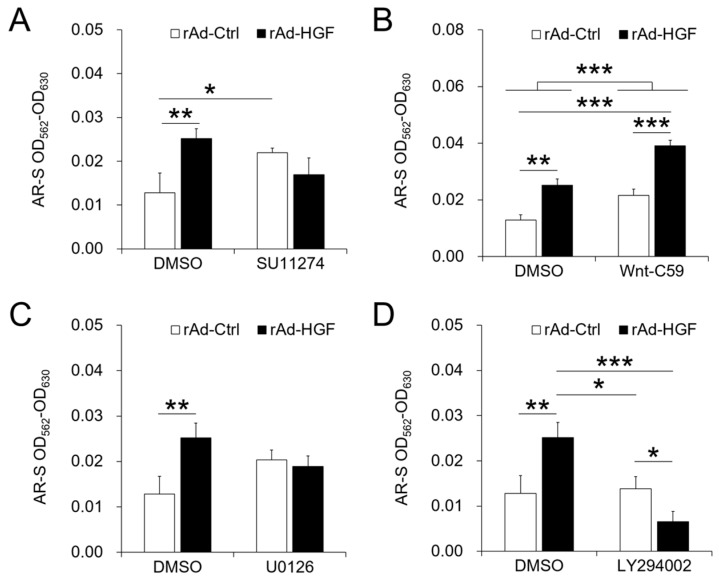
Roles of signaling pathways in osteogenesis of hi-BMSCs infected with recombinant adenoviruses. Human BMSCs were cultured in the osteogenic differentiation medium and treated with signal pathway inhibitors or DMSO before infection. Two weeks later, cell osteogenesis was detected using AR-S staining (**A**–**D**). * *p* < 0.05, ** *p* < 0.01, *** *p* < 0.001. The experiment has been replicated three times with similar results, and the representative results were shown.

Subsequently, the roles of above signaling pathways in hBMSC osteogenic differentiation were examined by treatments with the corresponding inhibitors. The degree of osteogenic differentiation was also assessed by the concentration of AR-S stained in the calcium nodules that formed by the hBMSCs. The results in DMSO-treated cells clearly indicated that the pro-osteogenic effects induced by rAd-HGF infection were significantly enhanced compared with that of rAd-Ctrl-infected cells (*p* < 0.01) (Figure 5A–D). 

Interestingly, treatment with SU11274 promoted osteogenic differentiation of cells infected with rAd-Ctrl when compared with DMSO-treated and rAd-Ctrl-infected cells (*p* < 0.05). Such promotion resulting from inhibition of c-MET could not be observed again when cells were infected with rAd-HGF. However, inhibition of c-MET led to the loss of pro-osteogenic effects in hBMSCs with rAd-HGF infection compared with DMSO-treated and rAd-HGF-infected cells. Together, the above results indicated that HGF-induced c-MET activation contributes to hBMSC osteogenic differentiation (Figure 5A). 

Considering the ineffectiveness of the WNT signaling pathway on hBMSC proliferation observed above, its function on the osteogenic differentiation is worth determining. Strikingly, in both DMSO-treatment and Wnt-C59 treatment groups, rAd-HGF infection significantly promoted osteogenic differentiation compared with rAd-Ctrl infection (*p* < 0.001), which demonstrated the anti-osteogenic role of the WNT signaling pathway in hBMSCs. Further, the osteogenic degree of the Wnt-59-treatment group was significantly enhanced compared with the DMSO-treatment group (Figure 5B). This finding suggested that the WNT signaling pathway is a critical anti-osteogenic regulator for the osteogenic differentiation of hBMSCs induced by HGF, and inhibition of the WNT pathway by Wnt-C59 may be a promising way to promote hBMSC osteogenesis (Figure 5B).

Meanwhile, consistent with the well-known function of the ERK1/2 pathway in promoting cell proliferation, our data indicated that inhibition of the ERK1/2 signaling has no significant effects on hBMSC osteogenic differentiation (Figure 5C).

Interestingly, inhibition of the PI3K/AKT pathway in rAd-Ctrl-infected cells by LY294002 did not suppress the osteogenic differentiation compared with the same cells treated with DMSO. However, in the case of LY294002 treatment, more significant suppression in osteogenic differentiation was observed in cells with rAd-HGF infection (*p* < 0.05), which is as an even more obvious situation when compared with DMSO-treated hBMSCs infected with rAd-HGF (*p* < 0.001). This finding suggested the important pro-osteogenic roles of PI3K/AKT pathway in rAd-HGF-induced hBMSC osteogenic differentiation (Figure 5D).

## 3. Discussion

BMSC adoptive cell therapy is a promising approach for ANFH. As a self-secreted cytokine, pleiotropic HGF has been demonstrated to play important roles in BMSC activity. In this study, the MOI-dependent effects of rAd-HGF infection for HGF expression on hBMSC proliferation and osteogenic differentiation have been explored, while the roles of c-MET-related signaling pathways are also investigated. Our results indicated that higher MOIs of rAd-HGF (MOI = 250 or MOI = 1250) not only significantly promoted HGF expression, but also played stronger roles to enhance hBMSC proliferation and osteogenic differentiation than rAd-Ctrl and low MOIs of rAd-HGF (MOI = 10 or MOI = 50) did. As MOI 250 showed the strongest effects in producing HGF and promoting both proliferation and osteogenesis of hBMSCs, it was selected for further evaluating the influences of HGF receptor c-MET-related signaling pathways in hBMSC proliferation and osteogenesis. Interestingly, inhibitor-treatment experiments showed that the WNT pathway plays anti-osteogenic roles while the PI3K/AKT pathway is pro-osteogenic for hBMSCs.

Signaling pathways play complex roles in cell bioactivity, also in that of hBMSCs in osteogenic differentiation environment as we demonstrated in this study. As the receptor of HGF on BMSCs, c-MET suppressed by SU11274 treatment significantly inhibited the proliferation of cells infected with both recombinant adenoviruses compared with DMSO-treated cells, and that of cells infected with rAd-HGF compared with cells infected with rAd-Ctrl, indicating that HGF promotes hBMSC proliferation through activating c-MET signaling. In addition, as expected, the ERK1/2 pathway activated by HGF functioned as a remarkable enhancing factor to promote hBMSC proliferation in the osteogenic differentiation condition. On the contrary, WNT signaling has been reported to regulate transcription of multiple genes, including the RUNX2 which promotes BMSC osteogenic differentiation [18]. For this reason, in this study, the WNT pathway was supposed to enhance BMSC osteogenic differentiation and possibly inhibit BMSC proliferation. However, when treated with Wnt-C59 to inhibit the WNT signaling, no effects on cell proliferation were observed when compared the DMSO-treated cells and the inhibitor-treated cells infected with the same recombinant adenovirus. The only difference appeared between Wnt-C59-treated and rAd-HGF-infected cells and DMSO-treated and rAd-Ctrl-infected cells (*p* < 0.05). These results indicated that the WNT pathway exerted no effects in hBMSC proliferation in the osteogenic medium. Similarly, the PI3K/AKT pathway also showed few effects on cell proliferation. These observations revealed that the c-MET and the ERK1/2 signaling pathways are involved in promotion of hBMSC proliferation in the osteogenic differentiation medium. However, rAd-HGF infection could inhibit these two pathways to suppress hBMSC proliferation under this condition.

It is well known that cell proliferation and differentiation are two opposite biological activities and regulated by different but maybe interactive signaling pathways. In this study, our results indicated that c-MET mainly promoted proliferation of hBMSCs. However, at the same time, inhibiting c-MET eliminated the promotive effects of rAd-HGF infection on hBMSC osteogenesis, indicating that c-MET also contributed to hBMSC differentiation. However, when cells were infected with rAd-Ctrl, treatment with SU11274 could greatly promote cell osteogenesis. Because BMSCs could self-secret HGF, this result suggested that the regulatory effects of c-MET were obvious at low dose of HGF, thus inhibiting c-MET not only inhibited cell proliferation, but also enhanced hBMSC osteogenic differentiation induced by self-secreted HGF. Meanwhile, consistent with the activity to promote cell proliferation, the ERK1/2 pathway exerted few effects on osteogenesis. These results indicated that the two cellular activities, proliferation and differentiation, indeed are opposite to each other. Interestingly, inhibition of the WNT pathway significantly promoted cell osteogenesis, contrary to the previous report on its promotive role in osteogenesis [18]. When rAd-HGF-infected hBMSCs were treated with Wnt-C59, the osteogenic degree was significantly higher than rAd-Ctrl-infected cells treated with DMSO (*p* < 0.001). Meanwhile, rAd-HGF-infected cells treated with DMSO showed comparable osteogenic degree when compared with rAd-Ctrl-infected cells treated with Wnt-C59, which suggested the HGF may be an inhibitory factor of the WNT pathway to promote hBMSC osteogenesis. In the meantime, as expected, our results indicated that the PI3K/AKT pathway indeed promoted hBMSC osteogenic differentiation. Treatment with LY294002 could greatly suppress the level of hBMSC osteogenesis elevated by rAd-HGF infection. Furthermore, similar osteogenic degrees between rAd-Ctrl-infected cells treated with DMSO and rAd-HGF-infected cells treated with LY294002 indicated that LY294002 could counteract the pro-osteogenic effects of the PI3K/AKT pathway after HGF activation. In summary, in rAd-HGF-infected hBMSCs, HGF induced activation of the PI3K/AKT pathway and inhibition of the WNT pathway functioned together to promote cell osteogenesis.

Here, we demonstrated the MOI-dependent effects of rAd-HGF on HGF expression and the proliferation and osteogenesis of hBMSCs, as well as the roles of various signaling pathways in rAd-HGF-mediated hBMSC proliferation and osteogenesis. Besides the positive regulatory roles of rAd-HGF with MOI = 250 in osteogenesis of hBMSCs, results of pathway-specific inhibitor treatments also demonstrated that the WNT pathway plays anti-osteogenic roles while the PI3K/AKT pathway is pro-osteogenic for hBMSCs. Our finding suggest that combinatory application of high MOI rAd-HGF (MOI = 250) with inhibitor of WNT pathway, like Wnt-C59, is a promising modality for the better application of HGF gene-modified hBMSCs in ANFH therapy.

## 4. Materials and Methods 

### 4.1. Cell Cultures

Human bone marrow mesenchymal stem cells (hBMSCs) were bought from Cyagen Biosciences Inc., (Goleta, CA, USA). Informed consent was obtained in accordance with the Declaration of Helsinki and the Institutional Review Board of the Southern Medical University (approval number SMU-2015123, 2016.01.10). Cells were cultured at 37 °C, 5% CO_2_, in the Human Mesenchymal Stem Cell Basal Medium complemented with 10% of the qualified fetal bovine serum (FBS), 1% Penicillin-streptomycin and 1% Glutamine (all from Cyagen Biosciences Inc.). The potency of osteogenic, chondrogenic, and adipogenic differentiation has been confirmed before obtainment. Cells were infected with recombinant adenoviruses and the proliferation and osteogenic differentiation were assayed. In some experiments, cells were treated with signaling pathway inhibitors agents as indicated in the figure legends, including c-MET inhibitor SU11274 (10 μM), WNT inhibitor Wnt-C59 (2 μM), ERK1/2 inhibitor U0126 (10 μM), PI3K/AKT inhibitor LY294002 (10 μM) (all from Selleckchem, Houston, TX, USA), and DMSO (Sigma-Aldrich, St. Louis, MO, USA) was used as the negative control.

### 4.2. Preparation of Recombinant Adenoviruses

Recombinant adenovirus carrying HGF gene (rAd-HGF) and empty adenovirus (rAd-Ctrl) were constructed, packaged, purified, and the titer was detected as described previously [21]. Prepared adenoviruses were aliquoted and stored at −80 °C before use.

### 4.3. ELISA

The supernatants of hBMSCs following treatments were collected and centrifuged at 3000× *g*, 4 °C for 5 min. The obtained supernatants were stored at −80 °C before assays. Secretion of HGF by hBMSCs were detected using ELISA kits (ExCell Bio, Shanghai, China) according to the protocols provided by the manufacturers. Considering the self-secretion of HGF by BMSCs, the results were deducted from the background. 

### 4.4. Cell Viability

Human BMSCs were inoculated at 2.5–4 × 10^4^/cm^2^ in 96-well plates (ExCell Bio) and cultured overnight. Cells were treated as indicated in the figure legends and cell viability was assayed at indicated time using WST-8 method (Cell Counting Kit-8; Dojindo, Kumamoto, Japan) according to the manufacturer’s instructions.

### 4.5. Osteogenic Differentiation and Calcium Accumulation Assay

Human BMSCs were inoculated at 2.5–4 × 10^4^/cm^2^ in 24-well plates. When the culture reached 100% confluence, the medium was changed to the Human Mesenchymal Stem Cell Osteogenic Differentiation Basal Medium complemented with 10% FBS, 1% Penicillin-streptomycin, 1% Glutamine, 0.2 mM ascorbate, 10 mM β-glycerophosphate, and 0.1 μM dexamethasone (all from Cyagen Biosciences Inc.). Media were changed every two days and the cells were cultured for 14–21 days. The calcium accumulation was assessed using alizarin red sulfate (AR-S; Cyagen Biosciences Inc.) staining and quantified as described previously [12].

### 4.6. Western Blot

At different time post treatments as indicated in the legends, hBMSCs were lysed using RIPA lysis buffer containing 10% protease inhibitor complex (Roche Applied Science, Mannheim, Germany), 10% PhosSTOP Phosphatase Inhibitor Cocktail (Roche) and 1 μM DL-Dithiothreitol (Sigma-Aldrich). Western blot was performed using following antibodies: phosphorylated-c-Met (D26; 1:2000), c-Met (25H2; 1:2000), phosphorylated-Akt (p-Akt) (D9E; 1:2000), Akt (C67E7; 1:2000), ERK2 (C-14; 1:2000), phosphorylated-ERK1/2 (p-ERK1/2) (E-4; 1:1000) (Cell Signaling Technology, Inc., Beverly, MA, USA), non-phospho (active)-β-catenin (D2U8Y; 1:1000), GAPDH (1:2000; Zhongshan Goldenbridge Biotechnology Co., Ltd., Beijing, China), and appropriate horseradish peroxidase–conjugated secondary antibodies (1:5000; Zhongshan Goldenbridge). The SuperSignal West Pico Chemiluminescent Substrate (Thermo Fisher Scientific Inc., Waltham, MA, USA) was used to develop the membranes according to manufacturer’s instructions. Results were observed and obtained using FluorChem Q Multi-Functional Imaging and Analysis System (ProteinSimple Ltd., San Jose, CA, USA).

### 4.7. Statistical Analysis

Data are expressed as the mean ± SD. The statistical significance was determined using one-way ANOVA. Post hoc multiple comparisons were performed using least significant difference or Dunnett’s T3. Differences with *p* < 0.05 were statistically significant. All statistical analyses were performed with SPSS statistical software version 16.0 (SPSS, Chicago, IL, USA).

## Supplementary Materials

Supplementary materials can be found at http://www.mdpi.com/1422-0067/19/3/734/s1.
