# Supplementary material for: The Multiplicity of Infection-Dependent Effects of Recombinant Adenovirus Carrying HGF Gene on the Proliferation and Osteogenic Differentiation of Human Bone Marrow Mesenchymal Stem Cells
Source: Int J Mol Sci. 2018 Mar 5;19(3):734. doi: 10.3390/ijms19030734 (PMC5877595; doi:10.3390/ijms19030734)
Supplement: Supplementary file 1 [file ijms-19-00734-s001.pdf]

International Journal of
Molecular Sciences

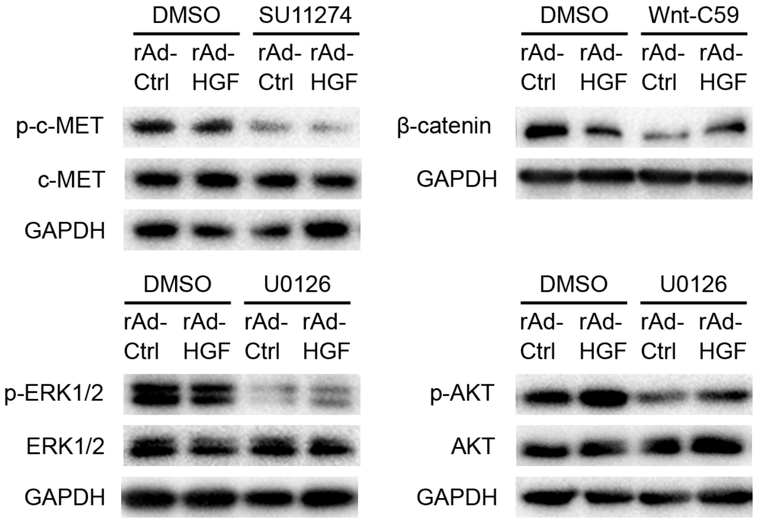

Supplemental Figure S1. Inhibitory effects of inhibitors on corresponding signaling pathways. Human BMSCs were cultured in the osteogenic differentiation medium and treated with various signal pathway inhibitors or DMSO followed by infection with recombinant adenoviruses. Three days later, the inhibitory effects on the various signaling pathways by their corresponding inhibitors have been confirmed using Western blot analysis. The experiment has been replicated three times with similar results, and the representative results were shown.

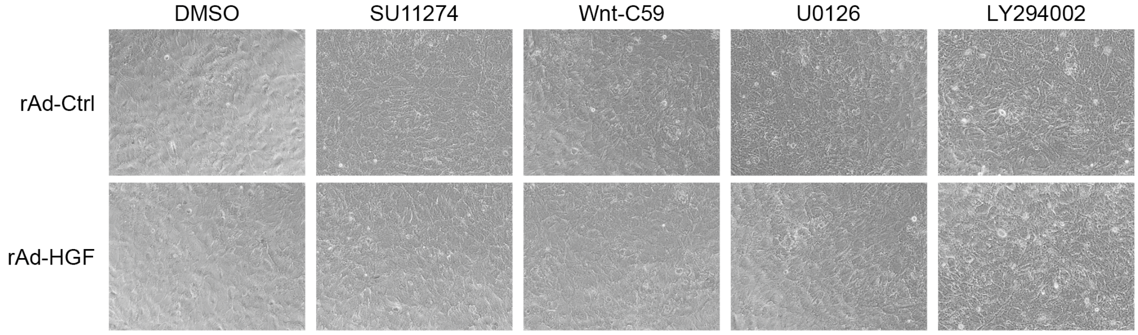

Supplemental Figure S2. Statuses of hBMSCs infected with recombinant adenoviruses combined with inhibitor treatment. Human BMSCs were cultured in the osteogenic differentiation medium and treated with various signal pathway inhibitors or DMSO followed by infection with recombinant adenoviruses. Three days later, the cell statuses were observed under the microscope, and the representative results were shown.
